# Joint Hypermobility in Paediatric Acute-Onset Neuropsychiatric Syndrome—A Preliminary Case-Control Study

**DOI:** 10.3389/fpsyt.2021.797165

**Published:** 2021-12-03

**Authors:** Susanne Bejerot, Ulrika Hylén, Martin Glans, Eva Hesselmark, Mats B. Humble

**Affiliations:** ^1^Faculty of Medicine and Health, University Health Care Research Center, Örebro University, Örebro, Sweden; ^2^School of Medical Sciences, Örebro University, Örebro, Sweden; ^3^Centre for Psychiatry Research, Department of Clinical Neuroscience, Karolinska Institutet, Stockholm, Sweden; ^4^Stockholm Health Care Services, Region Stockholm, CAP Research Center, Stockholm, Sweden

**Keywords:** postural orthostatic tachycardia syndrome, paediatric acute-onset neuropsychiatric syndrome, psychiatry, comorbidity, joint hypermobility, PNISSI

## Abstract

**Background:** Individuals with generalised joint hypermobility (GJH, present in 10–20% of the general population) are at increased risk of being diagnosed with a range of psychiatric and rheumatological conditions. It is unknown whether Paediatric acute-onset neuropsychiatric syndrome (PANS), characterised by childhood onset obsessive-compulsive disorder or restricted eating and typically associated with several comorbid neuropsychiatric symptoms, is associated with GJH. It is also unknown whether extensive psychiatric comorbidity is associated with GJH.

**Method:** This is a case-control study including 105 participants. We compared three groups: Individuals with PANS, individuals with other mental disorders and healthy controls. Joint mobility was assessed with the Beighton scoring system, psychiatric comorbidity with the M.I.N.I. or MINI-KID interview and symptoms of PANS with the PsychoNeuroInflammatory related Signs and Symptoms Inventory (PNISSI).

**Results:** Hypermobility was similar across groups, and high rates of psychiatric comorbidity was not associated with higher Beighton scores.

**Conclusion:** Although GJH is associated with several psychiatric conditions, such as ADHD and anxiety, this does not seem to be the case for PANS according to this preliminary study.

## Introduction

Generalised joint hypermobility (GJH) is present in 10–20% of the normal population, although the rate may vary to a considerable extent depending on the applied cutoff scores of the measure (1). Individuals with GJH are at increased risk of being diagnosed with a range of psychiatric disorders and rheumatological conditions (2–4). GJH has specifically been associated with Postural Orthostatic Tachycardia Syndrome (POTS); reportedly, more than 50% of subjects with joint hypermobility have both conditions (5). POTS in turn has been observed in individuals with paediatic acute-onset neuropsychiatric syndrome (PANS) (Bejerot S, unpublished data). PANS is an umbrella term that includes Paediatric autoimmune neuropsychiatric disorder associated with streptococcus (PANDAS). PANS is characterised by an abrupt onset of obsessive-compulsive disorder or restricted eating and typically involves multiple psychiatric and neurological symptoms including tics (6). Inflammation is suggested to play a role in the pathophysiology of both PANS and POTS, although the purported immunological mechanisms are still unclear (7, 8). Moreover, neuropsychiatric symptoms such as fatigue, anxiety, panic, fears, problems with concentration and memory, and sleep disturbance are common across these diagnoses (6, 9, 10). Almost 80% of Swedish children with PANS present anxiety at onset, and 43% present hyperactivity (11). Notably, anxiety and Attention-Deficit/Hyperactivity Disorder (ADHD) are both associated with GJH (12, 13). According to a conference report, several individuals suffer from both POTS and PANS, in addition to GJH (14).

The primary aim of the present study was to investigate if GJH is overrepresented in individuals with PANS. The secondary aim was to investigate if multiple psychiatric comorbidities are associated with joint hypermobility in individuals with mental disorders.

## Methods

### Study Design

This is a case–control study that compares three groups: Individuals with interview-confirmed PANS, individuals with other mental disorders, and healthy controls.

### Participants and Procedures

**Figure 1:**
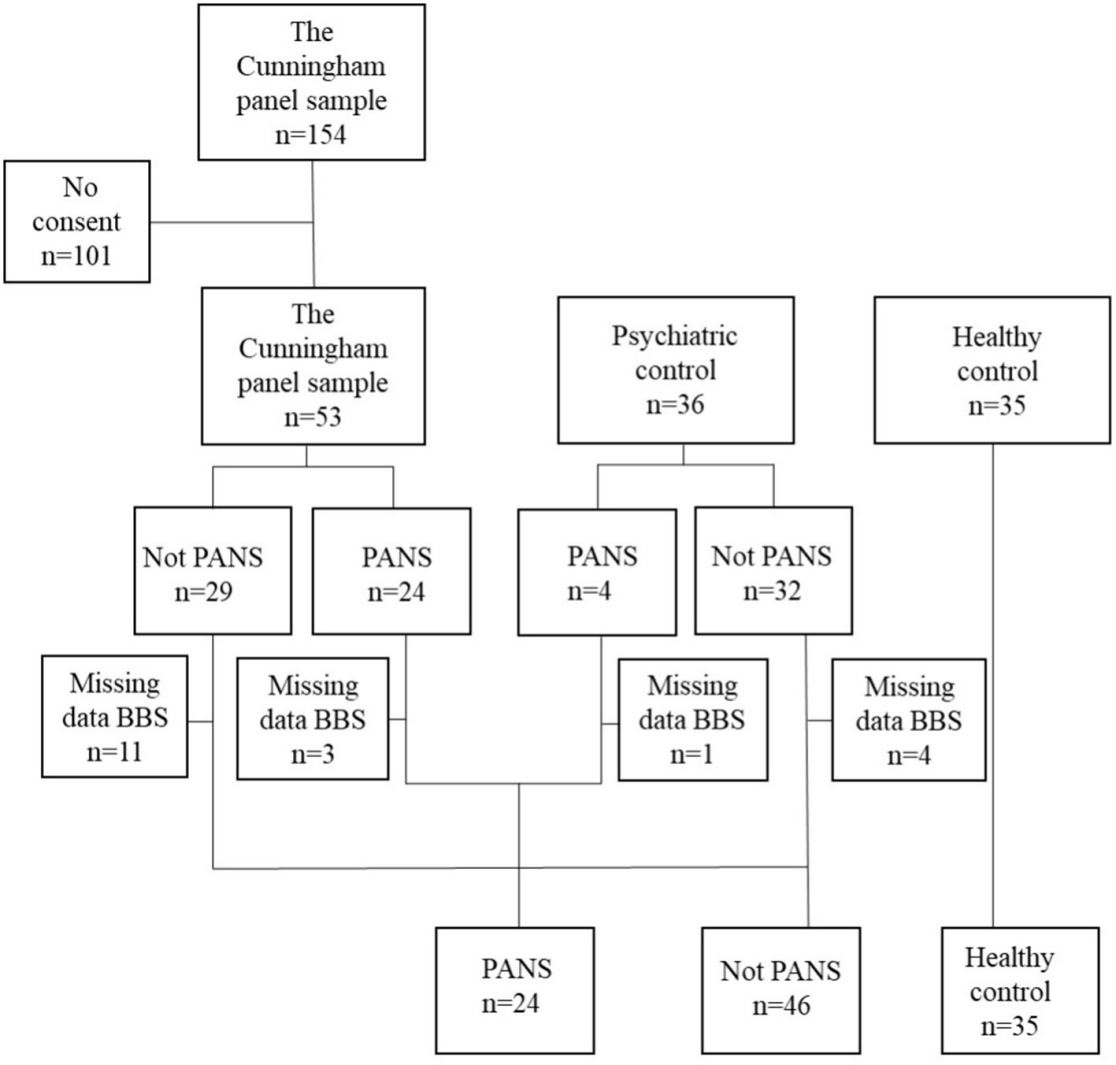
Flowchart of study participants.

Participants with PANS were enrolled through Wieslab, the Swedish laboratory that, at the time, provided a blood test (the Cunningham Panel) allegedly associated with PANS (15, 16). All 154 individuals who had taken the panel prior to June 2014 were invited. 53 individuals consented to participate in a study that investigated the validity of the Cunningham panel. The participants went through a comprehensive psychiatric assessment, for details, see Hesselmark and Bejerot (16). A total of 28 participants met formal PANS criteria and were assessed with the Beighton scoring system (BSS), thus constituting the PANS-sample of this study. The participants who did not meet PANS-criteria were enrolled in this study as psychiatric controls (*n* = 18). To broaden the psychiatric comparison group of individuals with psychiatric disorder we advertised for volunteers at a psychiatric clinic located in Örebro, Sweden. Inclusion criteria for this sample were to have a psychiatric disorder requiring specialist care and not fulfilling the diagnostic criteria for PANS. PANS was ruled out using the Psychoneuroinflammatory related signs and symptoms inventory, PNISSI. PNISSI is a comprehensive structured clinical interview based on a similarly comprehensive collateral informant questionnaire that is filled out by an informant prior to the clinical visit (17) (see Supplementary Material). This Örebro sample, examined with the BBS (*n* = 28), was assessed by the same senior psychiatrist (SB) or a senior child and adolescent psychiatrist. In total, 46 individuals constitute the psychiatric control sample in the present study (see Figure 1).

Finally, healthy controls (*n* = 35) matched to the PANS group by age, were also enrolled in Örebro during May and June 2018. They were recruited by convenience sampling from children of health-care staff and university employees. These participants were examined by a board-certified nurse (UH), trained in assessment of GJH. Mental illness was ruled out by asking each participant if they ever had any psychiatric disorder, i.e., the Mini International Neuropsychiatric Interviews (M.I.N.I. and MINI-KID) were not applied among the healthy controls.

All participants were interviewed on one occasion, either at a psychiatric facility or in their homes. A parent or a caretaker assisted if the participants was unable to complete the interview independently. Exclusion criteria for all participants in the present study were missing information on joint hypermobility, age ≥40 years and not speaking Swedish.

### Assessments

Generalised joint hypermobility (GJH) was examined with the Beighton scoring system (BSS) which is a physical test applied by a clinician. BSS gives no indication of the degree of hypermobility, merely an expression of the widespread nature of its distribution. The BSS involves examining 9 joints (4 bilateral and 1 unilateral) as follows: (1) Passive dorsiflexion and hyperextension of the fifth metacarpophalangeal joint beyond 90°; (2) Passive apposition of the thumb to the flexor aspect of the forearm; (3) Passive hyperextension of the elbow beyond 10°; (4) Passive hyperextension of the knee beyond 10°; (5) Active forward flexion of the trunk with the knees fully extended so that the palms of the hands rest flat on the floor. Each joint identified with hypermobility is counted as 1 point, with total scores ranging from 0 to 9 (18). A score of ≥5 is defined as GJH in people younger than 50 years (19). For children, a score of 5 in the BSS does not necessarily imply GJH. International Consortium on the Ehlers Danlos syndromes (EDS) proposed a cut-off score of ≥6 for diagnosis of GJH in pre-pubertal children and adolescents (19, 20). Other signs and symptoms suggestive of Ehlers-Danlos syndrome, e.g., chronic musculoskeletal pain, velvety skin, hernias/prolapses, Marfanoid habitus, and heart malformations were not assessed in this study, nor did we assess participants for POTS. The BSS assessment was performed by three different assessors.

The Mini International Neuropsychiatric Interview (M.I.N.I., version 6) is a structured screening interview for assessing multiple present and previous psychiatric diagnoses in adults (21). The MINI-KID (version 6) is adapted for children (22). Because the MINI-KID includes several items not included in the M.I.N.I. for adults, we added several MINI-KID modules (anxiety, specific phobias, Tourette syndrome/tics, ADHD, conduct disorder, and oppositional defiant disorder) in the adult interview.

### Statistics

All statistical analyses were conducted using SPSS (version 27). In order to test if the three groups had similar sex distribution the chi-squared tests for categorical variables was completed. Mann-Whitney *U* test was performed in analysis of number of diagnoses in the PANS vs. psychiatric control group. For age and BSS scores the non-parametric Kruskal-Wallis test for independent variables was used. For the correlation between BSS scores and number of psychiatric diagnoses, the Spearman's rho test was performed.

### Ethics

All procedures were approved by the Regional Ethics Review Board of Stockholm (2014/551-31/2; 2014/1711-32; 2015/964-31, 2016/2121-32; 2018/404-32). All study participants and/or legal guardians granted informed consent.

## Results

A total of 105 participants were assessed with M.I.N.I. and the BSS, 28 with PANS, 46 psychiatric controls and 35 healthy controls. The healthy controls were younger compared to the psychiatric controls. Psychiatric comorbidity was prevalent in both the PANS and the psychiatric control group (see Table 1).

**Table 1:** Demographics of participants at the time of the interview.

	PANS^*^	**Psychiatric control**	**Healthy control**	**Comparison between groups**
	***n =* 24**	***n =* 46**	***n =* 35**	**Statistics**
**General parameters**				
Sex, m/f	11/13	22/24	23/12	*C^2^*(2) = 3.3; *p* = 0.20
Age, median (range)	12.5 (8–36)	17.5 (6–40)	14 (4–33)	H(2) = 8.6; *p* = 0.014
Number of diagnoses, median (range)^**^	3.0 (1–11)	5.00 (1–13)	0	U = 621.5; *p* = 0.39
Beighton Score, median (range)	2.0 (0–9)	2.0 (0–7)	2.0 (0–7)	H(2) = 0.31; *p* = 0.86
Beighton Score ≥ 5, *n* (%)	4 (17)	9 (20)	5 (14)	*C^2^*(2) = 0.40; *p* = 0.82
Beighton Score ≥ 6, *n* (%)	3 (13)	5 (11)	3 (9)	*C^2^*(2) = 0.25; *p* = 0.88
**Psychiatric diagnoses**, ***n*** **(%)**				
Autism spectrum disorder	4 (17%)	18 (39%)		
ADHD	11 (46%)	25 (54%)		
Conduct disorder	0	2 (4%)		
Oppositional disorder	5 (21%)	16 (35%)		
Tourette's and tic disorder	13 (54%)	12 (26%)		
Obsessive compulsive disorder	17 (71%)	21 (46%)		
Generalised anxiety disorder	9 (38%)	21 (46%)		
Social anxiety disorder	3 (13%)	15 (32%)		
Panic disorder^a^	6 (25%)	14 (30%)		
Agoraphobia	3 (13%)	10 (22%)		
Anorexia nervosa	1 (4%)	0		
Bulimia nervosa	0	2 (4%)		
Depressive disorders^a^	17 (71%)	26 (57%)		
Bipolar and related disorders^a^	3 (13%)	3 (7%)		
Trauma related disorders	1 (4%)	3 (7%)		
Schizophrenia spectrum disorders^a^	9 (38%)	11 (24%)		

*PANS, paediatric acute-onset neuropsychiatric syndrome; ADHD, Attention-Deficit/Hyperactivity Disorder.*

* *Lifetime paediatric acute-onset neuropsychiatric syndrome (PANS).*

** *Comparisons are made between PANS and psychiatric controls.*

a *Lifetime diagnosis*.

Mean Beighton score (±S.D.) was 2.25 (2.5) in the PANS group, 2.33 (2.1) in psychiatric controls, and 2.09 (2.1) in healthy controls (n.s. between groups). Eleven participants reached a BSS score of ≥6 (n.s.). Pairwise Mann-Whitney tests between the PANS group and the two control groups were likewise non-significant.

**Figure 2:**
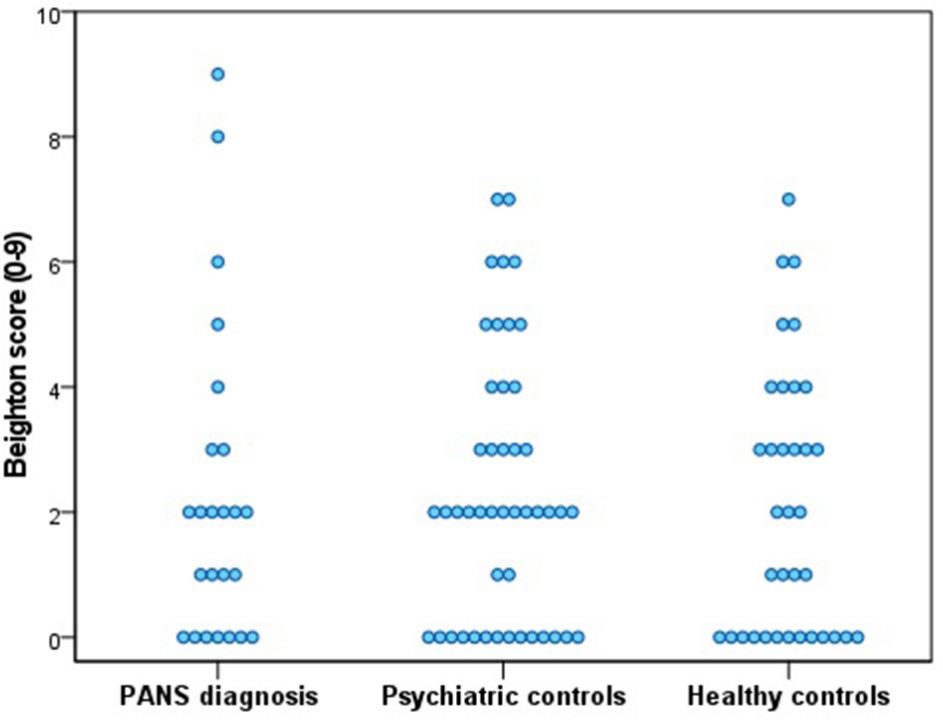
Individual Beighton scores of all participants.

We found no significant correlations in the collapsed PANS and psychiatric control group between number of comorbid psychiatric diagnoses, and the Beighton score (Spearman's rho = −0.25; *p* = 0.84) (see Figure 2).

## Discussion

The primary aim of this study was to determine if GJH is associated with PANS, in line with the association reported between POTS and GJH (23, 24). In our small sample of individuals with PANS we could not find any such association, nor could we find an overrepresentation of GJH among controls with mental disorder compared to a healthy control group. However, due to the small sample the results must be considered as preliminary.

Fourteen percent among the healthy controls were classified as hypermobile, which is slightly higher compared to other studies using the same BSS cut-off score (12, 20) and considerably higher than was reported in an exceptionally large study of British adolescents (1). However, Reuter and Fichthorn studied 416 American Caucasian white college students and reported 11.5% to be hypermobile (20). In a recent Swedish study on 417 adults, recruited from the normal population, 11% of the women, and 4.7% of the men were found to be hypermobile (12). Considering the low age in our sample compared to forementioned studies, a higher rate of GJH can be expected.

Notably, although joint hypermobility was similar among our three groups, the only two participants that scored 8 and 9 on the BSS belonged to the PANS group. This suggests that in a large sample of individuals with PANS a subpopulation with GJH may exist.

Our secondary aim was to investigate if multiple psychiatric comorbidities are associated with GJH which was not the case in this study. The comorbidity rate among the PANS group and the psychiatric controls was nevertheless high compared to what is generally reported in the literature. This may partly be explained by the fact that we applied an extension, consisting of childhood onset psychiatric diagnoses included in the MINI-KID, in both children and adults in the present study. Although MINI-KID is more comprehensive than the M.I.N.I., important psychiatric diagnoses such as Hoarding disorder and Body Dysmorphic Disorder are not included in neither of them, thus we may have missed additional comorbidities. Another possible reason for the high comorbidity rate was the recruitment base. We recruited participants from psychiatric clinics where people with severe forms of mental disorders are patients. People with less severe forms of psychiatric disorders are generally handled by general practitioners in Sweden, which was not our target group. Since the severity of a psychiatric disorder is known to be highly correlated with multiple comorbidities, this was an expected finding of our study. Possibly, individuals with mild forms or psychiatric disorders present other rates of GJH than our participants did. However, to this end, less than a handful psychiatric conditions are shown to be specifically associated to GJH.

## Limitations

There are undoubtedly several limitations to this study. First, the small sample with PANS including two participants with BBS scores of 8 and 9, raise the question whether we might have missed a hypermobile subgroup, and larger studies are warranted. Second, we were unsuccessful to recruit enough young children in the psychiatric control group, therefore their median age is higher compared to participants with PANS and healthy controls. The difference in age should however not influence our results, since children tend to have more flexible joints than adults (19). Third, three different clinicians assessed the participants; it would have been preferable if all participants had been examined by the same clinician. However, the BSS is rather simple to administrate, and all three assessors worked closely together and were jointly trained in the method. Fourth, in this study, we did not diagnose the new diagnostic entity known as generalised hypermobility spectrum disorder (G-HSD), defined by GJH in addition to chronic musculoskeletal pain and/or instability (25). This is due to the fact that we started the study on individuals with PANS already in 2014, prior to the introduction of G-HSD. Finally, due to the small sample size we cannot rule out a type II error.

## Conclusion

Although the onset of PANS is strongly associated with anxiety and ADHD, which are symptoms known to be associated with GJH, we did not find GJH to be more common among children and young adults with PANS. Neither was psychiatric comorbidity associated with GJH in the present study.

Future studies on PANS should ideally apply larger sample sizes and assess chronic musculoskeletal pain and/or instability to examine if G-HSD is an associated disorder.

## Data Availability Statement

The raw data supporting the conclusions of this article will be made available by the authors, without undue reservation.

## Ethics Statement

The studies involving human participants were reviewed and approved by Regional Ethics Review Board of Stockholm. Written informed consent to participate in this study was provided by the participants' legal guardian/next of kin.

## Author Contributions

SB and EH were responsible for the study concept, design, and data acquisition. UH assisted in the data collection on healthy controls. SB wrote the original draught. UH, MG, and SB performed analysis and interpretation of data. MH and MG conducted critical revision of the manuscript for intellectual content. All authors contributed to the manuscript and approved the submitted version.

## Funding

This research was funded by grants from the Swedish Research Council (SB Grant No: 523-2011-3646), Hjärnfonden (SB Grant No: FO2015-0191), Bror Gadelius Minnesfond (EH), Psykiatrifonden (EH), the Stockholm County Council (SB Grant No: PPG projects 20130671 and 20150150) and Bror Gadelius minnesfond (2019–2020) to MG. The funding sources had no influence over the study design, collection or interpretation of data or any other part of the research process.

## Conflict of Interest

The authors declare that the research was conducted in the absence of any commercial or financial relationships that could be construed as a potential conflict of interest.

## Publisher's Note

All claims expressed in this article are solely those of the authors and do not necessarily represent those of their affiliated organizations, or those of the publisher, the editors and the reviewers. Any product that may be evaluated in this article, or claim that may be made by its manufacturer, is not guaranteed or endorsed by the publisher.

## Supplementary Material

Data Sheet 1 PNISSI Part 1: Self-report/Collateral informant questionnaire.

Data Sheet 2 Clinician's interview.
