# Supplementary material for: Joint Hypermobility in Paediatric Acute-Onset Neuropsychiatric Syndrome—A Preliminary Case-Control Study
Source: Front Psychiatry. 2021 Dec 3;12:797165. doi: 10.3389/fpsyt.2021.797165 (PMC8678126; doi:10.3389/fpsyt.2021.797165)
Supplement: Data Sheet 1 — PNISSI Part 1: Self-report/Collateral informant questionnaire. [file Data_Sheet_1.PDF]

ID:

PNISSI self-assessment (you or your child)

This questionnaire includes questions where you can describe yourself and your problems. Your responses will provide a basis for further evaluation. If you complete the questionnaire on behalf of your child, then "you" refers to your child. Please bring this form to the appointment with your practitioner.

Patient's name: ....

Gender: ☐ Female ☐ Male Age: ....

Date of birth: ....

Today's date: ....

BACKGROUND

A. What are the main problems/symptoms now?

....

....

....

....

....

....

....

B. On a scale of 1–10 (where 10 is the most ill), how disabled or different do you feel now? (last few weeks) ....

C. On a scale of 1–10 (where 10 is the most ill), how disabled or different do you feel you were when you were at your very worst? ....

D. On a scale of 1–10, how disabled or different do others (i.e., other than family/relatives) consider you to be currently? (last few weeks).............

E. On a scale of 1–10, how disabled or different did others (i.e., other than family/relatives) consider you to be when you were at your very worst? ....

F. If you were to describe yourself, what words would describe you (3–5 descriptive words) before you became ill (before your problems started), and what words would describe you now?

BEFORE: ....

....

NOW: ....

....

G. Have you been given a diagnosis (physical or psychiatric) recently or in the past? ☐ Yes ☐ No

If yes, which diagnosis/diagnoses? ....

....

H. Have you been treated before (with medication or therapy or received support) to reduce the symptoms or consequences of your problems? ☐ Yes ☐ No, skip to question **N**.

a. If yes, which type of treatment and for what? ....

....

....

b. When and where (which hospital/clinic) did you receive the treatment? ....

....

c. Was the treatment helpful? ☐ Yes ☐ No ☐ Not sure

Comment:..

....

....

I. Are you presently being treated with medication, vitamins, or herbal remedies? ☐ Yes ☐ No ☐ Not sure

If yes, which ones? ....

....

....

J. If you have received drug treatment, did the medication have unexpected effects? For example, did you experience considerable adverse effects? ☐ Yes ☐ No ☐ Don't know ☐ Never received drug treatments

Comment:..

....

K. Do you currently have a medical doctor or other types of medical professionals? ☐ Yes ☐ No

If yes, with whom and where? ....

....

L. Are you currently receiving psychological treatment, or have you previously? ☐ Yes, previously ☐ Yes, currently ☐ No

a. If yes, what type? ....

b. From where (which hospital/clinic)? ....

c. Since approximately when? ....

M. Are you currently receiving professional support from social services or other forms of support, or have you previously? ☐ Yes, previously ☐ Yes, currently ☐ No

a. If yes, whar type? ....

b. From where (which hospital/clinic)? ....

c. Since approximately when?..

N. Have changes been made in your lifestyle to improve your health or to prevent your health from getting worse, such as a change in diet or other adjustments? ☐ Yes ☐ No

a. If yes, which changes? ....

b. If yes, have any of these changes led to improvement? ☐ Yes ☐ No ☐ Not sure

....

....

O. Has there been any improvement since you fell ill
(or since your problems started)? ☐ Yes ☐ No ☐ Not sure ☐ Cannot answer

a. If yes, what has improved? ....

....

....

b. How much of an improvement did you notice? ☐ Fully recovered ☐ Very much improved
☐ Much improved ☐ Moderately improved ☐ A little improved

c. Has the improvement been sustained? ☐ Yes ☐ No ☐ Not sure

....

....

....

P. Has deterioration occurred since you fell ill
(or since your problems started)? ☐ Yes ☐ No ☐ Not sure ☐ Cannot answer

a. If yes, which symptoms have become worse? ....

....

....

b. How much worse are you now? ☐ Infinitely worse ☐ Very much worse
☐ Much worse ☐ Moderately worse ☐ A little worse

_ _

Q. Do you have strengths or special abilities? ....

....

....

R. Were you born prematurely? ☐ Yes ☐ No ☐ Not sure

a. If yes, how many weeks early were you born (approximately)? .... ☐ Not sure

b. Were there any complications during pregnancy or child birth? ☐ Yes ☐ No ☐ Not sure

c. If yes, please describe ....

d. Incubator care, neonatal care? ☐ Yes ☐ No ☐ Not sure

e. If yes, for how long (approximately)? .... ☐ Not sure

f. Were you born by Caesarean section? ☐ Yes ☐ No ☐ Not sure

g. Were you breastfed? ☐ Yes ☐ No ☐ Not sure

h. If yes, for how long were you breastfed (approximately)? ....

i. During infancy (first year of life), did you have marked problems
regarding e.g. sleep, feeding, intense crying, or infections? ☐ Yes ☐ No ☐ Not sure

j. If yes, what sorts of problems? ....

....

k. Were you conceived through fertility treatments (e.g. IVF)? ☐ Yes ☐ No ☐ Not sure

S. How old were you the first time you were treated with antibiotics? (if not sure, try to estimate)? .... ☐ Never

T. Have you had repeated ear infections (otitis)? ☐ Yes ☐ No ☐ Not sure

a. Was an eardrum perforated, or were tubes inserted? ☐ Yes ☐ No ☐ Not sure

U. Have you had recurring tonsillitis (strep throat)? ☐ Yes ☐ No ☐ Not sure

V. Have you had scarlet fever, perianal streptococca dermatitis (perianal rash), or impetigo? ☐ Yes ☐ No ☐ Not sure

W. Have you had other recurrent skin infections? ☐ Yes ☐ No ☐ Not sure

X. Have you had urinary tract infections? ☐ Yes ☐ No ☐ Not sure

Y. Have you had any severe infections not listed above? ☐ Yes ☐ No ☐ Not sure

Z. If yes, where, please comment: ....

....

AA. Have you had an infected tooth or root canal filling? (besides common dental caries)? ☐ Yes ☐ No ☐ Not sure

BB. Has the gland behind your nose been surgically removed? ☐ Yes ☐ No ☐ Not sure

CC. Have your tonsils been surgically removed? ☐ Yes ☐ No ☐ Not sure

DD. Has an examination of your brain been performed? ☐ Yes ☐ No ☐ Not sure

a. If yes, which type of examination? ☐ Yes ☐ No ☐ Not sure

b. Where and when was it performed?? ....

....

EE. Have you been diagnosed with any of the following illnesses: epilepsy, autoimmune disorder, inflammatory disorder, hay fever, allergy, asthma, or immune deficiency? ☐ Yes ☐ No ☐ Not sure

a.If yes, which illness? ....

b. Was it considered severe? ☐ Yes ☐ No ☐ Not sure

Comment: ....

....

c. Which tests and/or assessments were used? ....

....

d. At what age, approximately, were you diagnosed? ....

e. Where (hospital/clinic) were you assessed? ....

FF. Have you undergone surgery or had general anesthesia? ☐ Yes ☐ No ☐ Not sure

a. If yes, what did you undergo surgery for? ....

b. When did you have surgery? ....

c. Where did you have the surgery (hospital/clinic)? ....

....

GG. Questions about difficulties in everyday life:

a. Are you currently (last few weeks) unable to attend school or work? ☐ Yes ☐ No

b. Do you have an assistant at school or support at home? ☐ Yes ☐ No

c. Do you have an assistant or caregiver at home? ☐ Yes ☐ No

d. Has a parent given up work in order to take care of you? ☐ Yes ☐ No ☐ Partly

e. Have you been granted a care allowance (or financial support for a disability)? ☐ Yes ☐ No

f. Do you isolate yourself in your home? ☐ Yes ☐ No ☐ Partly

g. Do you have difficulties in coping with things that others your age can do? ☐ Yes ☐ No ☐ Partly

h. Have you been bullied at school or at work? ☐ Yes ☐ No ☐ Not sure

---

HH. Below you will be asked about different problem areas that you may have. Each question contains four sub-questions: a, b, c, and d. These are related to how the problems started, how long they lasted, and whether they resulted in problems nowadays or previously. Please respond to the questions the best you can. If several alternatives apply, then you can mark several options. You are invited to provide examples. You may use an extra sheet of paper if needed.

---

1. Compulsive symptoms, such as compulsively repeating actions or having compulsive thoughts/fixed ideas, such as excessive hand washing, fear of dirt or germs, obsessions about death, fear of harming others, repeated checking, need or a feeling that everything must be "just so," or excessive hoarding (circle the appropriate description/s).

   ☐ Yes, always/as long as you can remember
   ☐ Yes, but it has occurred later (year? ....)
   ☐ Yes, in the past, but not now
   ☐ No, never
   ☐ Cannot be determined/Not applicable
   Please provide examples:

a. If the description is true, did it occur suddenly, or gradually?
☐ Suddenly ☐ Gradually ☐ Don't know

b. How long have you exhibited this behavior?
☐ Only occasionally ☐ Less than 3 months
☐ 3 months–2 years ☐ Several years ☐ It varies

c. Is this behavior currently (the past 2 weeks) causing any problems?
☐ A lot ☐ Somewhat ☐ A little
☐ None at all ☐ Don't know

d. Has this behavior caused any problems in the past?
☐ A lot ☐ Somewhat ☐ A little
☐ None at all ☐ Don't know

2. Abnormal eating behaviors, wanting to eat only a few select things, or eating too little or too much (circle the appropriate description/s).

   ☐ Yes, always/as long as you can remember
   ☐ Yes, but it has occurred later (year? ....)
   ☐ Yes, in the past, but not now
   ☐ No, never
   ☐ Cannot be determined/Not applicable
   Please provide examples:

   a. If the description is true, did it occur suddenly, or gradually?
      ☐ Suddenly ☐ Gradually ☐ Don't know
   b. How long have you exhibited this behavior?
      ☐ Only occasionally ☐ Less than 3 months
      ☐ 3 months–2 years ☐ Several years ☐ It varies
   c. Is this behavior currently (the past 2 weeks) causing any problems?
      ☐ A lot ☐ Somewhat ☐ A little
      ☐ None at all ☐ Don't know
   d. Has this behavior caused any problems in the past?
      ☐ A lot ☐ Somewhat ☐ A little
      ☐ None at all ☐ Don't know

---

3. Separation anxiety, such as fear of being away from family, e.g., afraid to sleep alone.

   ☐ Yes, always/as long as you can remember
   ☐ Yes, but it has occurred later (year? ....)
   ☐ Yes, in the past, but not now
   ☐ No, never
   ☐ Cannot be determined/Not applicable
   Please provide examples:

   a. If the description is true, did it occur suddenly, or gradually?
      ☐ Suddenly ☐ Gradually ☐ Don't know
   b. How long have you exhibited this behavior?
      ☐ Only occasionally ☐ Less than 3 months
      ☐ 3 months–2 years ☐ Several years ☐ It varies
   c. Is this behavior currently (the past 2 weeks) causing any problems?
      ☐ A lot ☐ Somewhat ☐ A little
      ☐ None at all ☐ Don't know
   d. Has this behavior caused any problems in the past?
      ☐ A lot ☐ Somewhat ☐ A little
      ☐ None at all ☐ Don't know

---

4. Depressed and sad, e.g., no will to live or shift in mood between being happy to being heartbroken (circle the appropriate description/s).

   ☐ Yes, always/as long as you can remember
   ☐ Yes, but it has occurred later (year? ....)
   ☐ Yes, in the past, but not now
   ☐ No, never
   ☐ Cannot be determined/Not applicable
   Please provide examples:

   a. If the description is true, did it occur suddenly, or gradually?
      ☐ Suddenly ☐ Gradually ☐ Don't know
   b. How long have you exhibited this behavior?
      ☐ Only occasionally ☐ Less than 3 months
      ☐ 3 months–2 years ☐ Several years ☐ It varies
   c. Is this behavior currently (the past 2 weeks) causing any problems?
      ☐ A lot ☐ Somewhat ☐ A little
      ☐ None at all ☐ Don't know
   d. Has this behavior caused any problems in the past?
      ☐ A lot ☐ Somewhat ☐ A little
      ☐ None at all ☐ Don't know

---

5. Easily irritated with yourself or others (circle the appropriate description/s).

☐ Yes, always/as long as you can remember
☐ Yes, but it has occurred later (year? ....)
☐ Yes, in the past, but not now
☐ No, never
☐ Cannot be determined/Not applicable

Please provide examples:

a. If the description is true, did it occur suddenly, or gradually?
☐ Suddenly ☐ Gradually ☐ Don't know

b. How long have you exhibited this behavior?
☐ Only occasionally ☐ Less than 3 months
☐ 3 months–2 years ☐ Several years ☐ It varies

c. Is this behavior currently (the past 2 weeks) causing any problems?
☐ A lot ☐ Somewhat ☐ A little
☐ None at all ☐ Don't know

d. Has this behavior caused any problems in the past?
☐ A lot ☐ Somewhat ☐ A little
☐ None at all ☐ Don't know

---

6. Have frequently and obviously irritated others on purpose and refused to comply with requests.

☐ Yes, always/as long as you can remember
☐ Yes, but it has occurred later (year? ....)
☐ Yes, in the past, but not now
☐ No, never
☐ Cannot be determined/Not applicable

Please provide examples:

a. If the description is true, did it occur suddenly, or gradually?
☐ Suddenly ☐ Gradually ☐ Don't know

b. How long have you exhibited this behavior?
☐ Only occasionally ☐ Less than 3 months
☐ 3 months–2 years ☐ Several years ☐ It varies

c. Is this behavior currently (the past 2 weeks) causing any problems?
☐ A lot ☐ Somewhat ☐ A little
☐ None at all ☐ Don't know

d. Has this behavior caused any problems in the past?
☐ A lot ☐ Somewhat ☐ A little
☐ None at all ☐ Don't know

7-8. Have been physically violent, hit others or oneself, or destroyed things or hurt oneself (circle the description/s).

☐ Yes, always/as long as you can remember
☐ Yes, but it has occurred later (year? ....)
☐ Yes, in the past, but not now
☐ No, never
☐ Cannot be determined/Not applicable

Please provide examples:

a. If the description is true, did it occur suddenly, or gradually?
☐ Suddenly ☐ Gradually ☐ Don't know

b. How long have you exhibited this behavior?
☐ Only occasionally ☐ Less than 3 months
☐ 3 months–2 years ☐ Several years ☐ It varies

c. Is this behavior currently (the past 2 weeks) causing any problems?
☐ A lot ☐ Somewhat ☐ A little
☐ None at all ☐ Don't know

d. Has this behavior caused any problems in the past?
☐ A lot ☐ Somewhat ☐ A little
☐ None at all ☐ Don't know

9. Changed in the way of being, appeared partly or fully as a different person after the onset of the illness (or when the problems started).

☐ Yes, always/as long as you can remember
☐ Yes, but it has occurred later (year? ....)
☐ Yes, in the past, but not now
☐ No, never
☐ Cannot be determined/Not applicable
Please provide examples:

a. If the description is true, did it occur suddenly, or gradually?
☐ Suddenly ☐ Gradually ☐ Don't know

b. How long have you exhibited this behavior?
☐ Only occasionally ☐ Less than 3 months
☐ 3 months–2 years ☐ Several years ☐ It varies

c. Is this behavior currently (the past 2 weeks) causing any problems?
☐ A lot ☐ Somewhat ☐ A little
☐ None at all ☐ Don't know

d. Has this behavior caused any problems in the past?
☐ A lot ☐ Somewhat ☐ A little
☐ None at all ☐ Don't know

---

10. Regression in development, acting or thinking as if one was younger than actual age or as a young child (circle the appropriate description/s).

☐ Yes, always/as long as you can remember
☐ Yes, but it has occurred later (year? ....)
☐ Yes, in the past, but not now
☐ No, never
☐ Cannot be determined/Not applicable
Please provide examples:

a. If the description is true, did it occur suddenly, or gradually?
☐ Suddenly ☐ Gradually ☐ Don't know

b. How long have you exhibited this behavior?
☐ Only occasionally ☐ Less than 3 months
☐ 3 months–2 years ☐ Several years ☐ It varies

c. Is this behavior currently (the past 2 weeks) causing any problems?
☐ A lot ☐ Somewhat ☐ A little
☐ None at all ☐ Don't know

d. Has this behavior caused any problems in the past?
☐ A lot ☐ Somewhat ☐ A little
☐ None at all ☐ Don't know

---

11. Difficulties at work/school because of such difficulties as remembering things, understanding, or concentrating (circle the appropriate description/s).

☐ Yes, always/as long as you can remember
☐ Yes, but it has occurred later (year? ....)
☐ Yes, in the past, but not now
☐ No, never
☐ Cannot be determined/Not applicable
Please provide examples:

a. If the description is true, did it occur suddenly, or gradually?
☐ Suddenly ☐ Gradually ☐ Don't know

b. How long have you exhibited this behavior?
☐ Only occasionally ☐ Less than 3 months
☐ 3 months–2 years ☐ Several years ☐ It varies

c. Is this behavior currently (the past 2 weeks) causing any problems?
☐ A lot ☐ Somewhat ☐ A little
☐ None at all ☐ Don't know

d. Has this behavior caused any problems in the past?
☐ A lot ☐ Somewhat ☐ A little
☐ None at all ☐ Don't know

12. Hypersensitive senses, such as hearing, seeing, touching, or smelling (circle the appropriate description/s).

☐ Yes, always/as long as you can remember
☐ Yes, but it has occurred later (year? ....)
☐ Yes, in the past, but not now
☐ No, never
☐ Cannot be determined/Not applicable
Please provide examples:

a. If the description is true, did it occur suddenly, or gradually?
☐ Suddenly ☐ Gradually ☐ Don't know

b. How long have you exhibited this behavior?
☐ Only occasionally ☐ Less than 3 months
☐ 3 months–2 years ☐ Several years ☐ It varies

c. Is this behavior currently (the past 2 weeks) causing any problems?
☐ A lot ☐ Somewhat ☐ A little
☐ None at all ☐ Don't know

d. Has this behavior caused any problems in the past?
☐ A lot ☐ Somewhat ☐ A little
☐ None at all ☐ Don't know

---

13. Seeing, hearing, or smelling things that others don't perceive (circle the appropriate description/s).

☐ Yes, always/as long as you can remember
☐ Yes, but it has occurred later (year? ....)
☐ Yes, in the past, but not now
☐ No, never
☐ Cannot be determined/Not applicable
Please provide examples:

a. If the description is true, did it occur suddenly, or gradually?
☐ Suddenly ☐ Gradually ☐ Don't know

b. How long have you exhibited this behavior?
☐ Only occasionally ☐ Less than 3 months
☐ 3 months–2 years ☐ Several years ☐ It varies

c. Is this behavior currently (the past 2 weeks) causing any problems?
☐ A lot ☐ Somewhat ☐ A little
☐ None at all ☐ Don't know

d. Has this behavior caused any problems in the past?
☐ A lot ☐ Somewhat ☐ A little
☐ None at all ☐ Don't know

---

14. Experiencing oneself (e.g., body parts) or others in one's surroundings as changed, or experiencing the surroundings themselves as changed (circle the appropriate description/s).

☐ Yes, always/as long as you can remember
☐ Yes, but it has occurred later (year? ....)
☐ Yes, in the past, but not now
☐ No, never
☐ Cannot be determined/Not applicable
Please provide examples:

a. If the description is true, did it occur suddenly, or gradually?
☐ Suddenly ☐ Gradually ☐ Don't know

b. How long have you exhibited this behavior?
☐ Only occasionally ☐ Less than 3 months
☐ 3 months–2 years ☐ Several years ☐ It varies

c. Is this behavior currently (the past 2 weeks) causing any problems?
☐ A lot ☐ Somewhat ☐ A little
☐ None at all ☐ Don't know

d. Has this behavior caused any problems in the past?
☐ A lot ☐ Somewhat ☐ A little
☐ None at all ☐ Don't know

15. Feeling that you're being followed or being watched, without any evidence to suggest such thoughts.

☐ Yes, always/as long as you can remember
☐ Yes, but it has occurred later (year? ....)
☐ Yes, in the past, but not now
☐ No, never
☐ Cannot be determined/Not applicable

Please provide examples:

a. If the description is true, did it occur suddenly, or gradually?
☐ Suddenly ☐ Gradually ☐ Don't know

b. How long have you exhibited this behavior?
☐ Only occasionally ☐ Less than 3 months
☐ 3 months–2 years ☐ Several years ☐ It varies

c. Is this behavior currently (the past 2 weeks) causing any problems?
☐ A lot ☐ Somewhat ☐ A little
☐ None at all ☐ Don't know

d. Has this behavior caused any problems in the past?
☐ A lot ☐ Somewhat ☐ A little
☐ None at all ☐ Don't know

16. Hold rigid poses, experience an extreme loss of motor skills, become shut in on yourself, become mute, or speak very little (circle the appropriate description/s).

☐ Yes, always/as long as you can remember
☐ Yes, but it has occurred later (year? ....)
☐ Yes, in the past, but not now
☐ No, never
☐ Cannot be determined/Not applicable

Please provide examples:

a. If the description is true, did it occur suddenly, or gradually?
☐ Suddenly ☐ Gradually ☐ Don't know

b. How long have you exhibited this behavior?
☐ Only occasionally ☐ Less than 3 months
☐ 3 months–2 years ☐ Several years ☐ It varies

c. Is this behavior currently (the past 2 weeks) causing any problems?
☐ A lot ☐ Somewhat ☐ A little
☐ None at all ☐ Don't know

d. Has this behavior caused any problems in the past?
☐ A lot ☐ Somewhat ☐ A little
☐ None at all ☐ Don't know

17. Altered gaze, dilated pupils, or looking terrified (circle the appropriate description/s).

☐ Yes, always/as long as you can remember
☐ Yes, but it has occurred later (year? ....)
☐ Yes, in the past, but not now
☐ No, never
☐ Cannot be determined/Not applicable

Please provide examples:

a. If the description is true, did it occur suddenly, or gradually?
☐ Suddenly ☐ Gradually ☐ Don't know

b. How long have you exhibited this behavior?
☐ Only occasionally ☐ Less than 3 months
☐ 3 months–2 years ☐ Several years ☐ It varies

c. Is this behavior currently (the past 2 weeks) causing any problems?
☐ A lot ☐ Somewhat ☐ A little
☐ None at all ☐ Don't know

d. Has this behavior caused any problems in the past?
☐ A lot ☐ Somewhat ☐ A little
☐ None at all ☐ Don't know

18. Worse in gymnastics or ball sports than average, difficult to learn to dance, or appear to have muscle weakness (circle the appropriate description/s).

☐ Yes, always/as long as you can remember
☐ Yes, but it has occurred later (year? ....)
☐ Yes, in the past, but not now
☐ No, never
☐ Cannot be determined/Not applicable
Please provide examples:

a. If the description is true, did it occur suddenly, or gradually?
☐ Suddenly ☐ Gradually ☐ Don't know

b. How long have you exhibited this behavior?
☐ Only occasionally ☐ Less than 3 months
☐ 3 months–2 years ☐ Several years ☐ It varies

c. Is this behavior currently (the past 2 weeks) causing any problems?
☐ A lot ☐ Somewhat ☐ A little
☐ None at all ☐ Don't know

d. Has this behavior caused any problems in the past?
☐ A lot ☐ Somewhat ☐ A little
☐ None at all ☐ Don't know

19. Poor handwriting, seem to avoid writing or drawing, or doing so differently (circle the appropriate description/s).

☐ Yes, always/as long as you can remember
☐ Yes, but it has occurred later (year? ....)
☐ Yes, in the past, but not now
☐ No, never
☐ Cannot be determined/Not applicable
Please provide examples:

a. If the description is true, did it occur suddenly, or gradually?
☐ Suddenly ☐ Gradually ☐ Don't know

b. How long have you exhibited this behavior?
☐ Only occasionally ☐ Less than 3 months
☐ 3 months–2 years ☐ Several years ☐ It varies

c. Is this behavior currently (the past 2 weeks) causing any problems?
☐ A lot ☐ Somewhat ☐ A little
☐ None at all ☐ Don't know

d. Has this behavior caused any problems in the past?
☐ A lot ☐ Somewhat ☐ A little
☐ None at all ☐ Don't know

20. Marked involuntary or unusual movements of the body or face or make noises such as beeps, grunts, or shouts (circle the appropriate description/s).

☐ Yes, always/as long as you can remember
☐ Yes, but it has occurred later (year? ....)
☐ Yes, in the past, but not now
☐ No, never
☐ Cannot be determined/Not applicable
Please provide examples:

a. If the description is true, did it occur suddenly, or gradually?
☐ Suddenly ☐ Gradually ☐ Don't know

b. How long have you exhibited this behavior?
☐ Only occasionally ☐ Less than 3 months
☐ 3 months–2 years ☐ Several years ☐ It varies

c. Is this behavior currently (the past 2 weeks) causing any problems?
☐ A lot ☐ Somewhat ☐ A little
☐ None at all ☐ Don't know

d. Has this behavior caused any problems in the past?
☐ A lot ☐ Somewhat ☐ A little
☐ None at all ☐ Don't know

21-22. Hold rigid poses, experience an extreme loss of motor skills, become shut in on yourself, become mute, or speak very little (circle the appropriate description/s).

☐ Yes, always/as long as you can remember
☐ Yes, but it has occurred later (year? ....)
☐ Yes, in the past, but not now
☐ No, never
☐ Cannot be determined/Not applicable
Please provide examples:

a. If the description is true, did it occur suddenly, or gradually?
☐ Suddenly ☐ Gradually ☐ Don't know

b. How long have you exhibited this behavior?
☐ Only occasionally ☐ Less than 3 months
☐ 3 months–2 years ☐ Several years ☐ It varies

c. Is this behavior currently (the past 2 weeks) causing any problems?
☐ A lot ☐ Somewhat ☐ A little
☐ None at all ☐ Don't know

d. Has this behavior caused any problems in the past?
☐ A lot ☐ Somewhat ☐ A little
☐ None at all ☐ Don't know

23. Urinary tract symptoms, such as needing to urinate frequently or wetting yourself (circle the appropriate description/s).

☐ Yes, always/as long as you can remember
☐ Yes, but it has occurred later (year? ....)
☐ Yes, in the past, but not now
☐ No, never
☐ Cannot be determined/Not applicable
Please provide examples:

a. If the description is true, did it occur suddenly, or gradually?
☐ Suddenly ☐ Gradually ☐ Don't know

b. How long have you exhibited this behavior?
☐ Only occasionally ☐ Less than 3 months
☐ 3 months–2 years ☐ Several years ☐ It varies

c. Is this behavior currently (the past 2 weeks) causing any problems?
☐ A lot ☐ Somewhat ☐ A little
☐ None at all ☐ Don't know

d. Has this behavior caused any problems in the past?
☐ A lot ☐ Somewhat ☐ A little
☐ None at all ☐ Don't know

24. Have bowel problems, such as stomach pain, constipation, or diarrhea (circle the appropriate description/s).

☐ Yes, always/as long as you can remember
☐ Yes, but it has occurred later (year? ....)
☐ Yes, in the past, but not now
☐ No, never
☐ Cannot be determined/Not applicable
Please provide examples:

a. If the description is true, did it occur suddenly, or gradually?
☐ Suddenly ☐ Gradually ☐ Don't know

b. How long have you exhibited this behavior?
☐ Only occasionally ☐ Less than 3 months
☐ 3 months–2 years ☐ Several years ☐ It varies

c. Is this behavior currently (the past 2 weeks) causing any problems?
☐ A lot ☐ Somewhat ☐ A little
☐ None at all ☐ Don't know

d. Has this behavior caused any problems in the past?
☐ A lot ☐ Somewhat ☐ A little
☐ None at all ☐ Don't know

25. Have aches or pains, such as headaches or muscle pain (circle the appropriate description/s).

☐ Yes, always/as long as you can remember
☐ Yes, but it has occurred later (year? ....)
☐ Yes, in the past, but not now
☐ No, never
☐ Cannot be determined/Not applicable

Please provide examples:

a. If the description is true, did it occur suddenly, or gradually?
☐ Suddenly ☐ Gradually ☐ Don't know

b. How long have you exhibited this behavior?
☐ Only occasionally ☐ Less than 3 months
☐ 3 months–2 years ☐ Several years ☐ It varies

c. Is this behavior currently (the past 2 weeks) causing any problems?
☐ A lot ☐ Somewhat ☐ A little
☐ None at all ☐ Don't know

d. Has this behavior caused any problems in the past?
☐ A lot ☐ Somewhat ☐ A little
☐ None at all ☐ Don't know

---

26. Sleep disturbances, such as problems in falling asleep, interrupted sleep, or odd behavior during sleep (circle the appropriate description/s).

☐ Yes, always/as long as you can remember
☐ Yes, but it has occurred later (year? ....)
☐ Yes, in the past, but not now
☐ No, never
☐ Cannot be determined/Not applicable

Please provide examples:

a. If the description is true, did it occur suddenly, or gradually?
☐ Suddenly ☐ Gradually ☐ Don't know

b. How long have you exhibited this behavior?
☐ Only occasionally ☐ Less than 3 months
☐ 3 months–2 years ☐ Several years ☐ It varies

c. Is this behavior currently (the past 2 weeks) causing any problems?
☐ A lot ☐ Somewhat ☐ A little
☐ None at all ☐ Don't know

d. Has this behavior caused any problems in the past?
☐ A lot ☐ Somewhat ☐ A little
☐ None at all ☐ Don't know

---

27. Have no energy, grow tired without obvious cause.

☐ Yes, always/as long as you can remember
☐ Yes, but it has occurred later (year? ....)
☐ Yes, in the past, but not now
☐ No, never
☐ Cannot be determined/Not applicable

Please provide examples:

a. If the description is true, did it occur suddenly, or gradually?
☐ Suddenly ☐ Gradually ☐ Don't know

b. How long have you exhibited this behavior?
☐ Only occasionally ☐ Less than 3 months
☐ 3 months–2 years ☐ Several years ☐ It varies

c. Is this behavior currently (the past 2 weeks) causing any problems?
☐ A lot ☐ Somewhat ☐ A little
☐ None at all ☐ Don't know

d. Has this behavior caused any problems in the past?
☐ A lot ☐ Somewhat ☐ A little
☐ None at all ☐ Don't know

---

28. Are hyperactive, have difficulty sitting still.

☐ Yes, always/as long as you can remember
☐ Yes, but it has occurred later (year? ....)
☐ Yes, in the past, but not now
☐ No, never
☐ Cannot be determined/Not applicable

Please provide examples:

a. If the description is true, did it occur suddenly, or gradually?
☐ Suddenly ☐ Gradually ☐ Don't know

b. How long have you exhibited this behavior?
☐ Only occasionally ☐ Less than 3 months
☐ 3 months–2 years ☐ Several years ☐ It varies

c. Is this behavior currently (the past 2 weeks) causing any problems?
☐ A lot ☐ Somewhat ☐ A little
☐ None at all ☐ Don't know

d. Has this behavior caused any problems in the past?
☐ A lot ☐ Somewhat ☐ A little
☐ None at all ☐ Don't know

---

29. Vision is affected or blurres (not fixable by glasses).

☐ Yes, always/as long as you can remember
☐ Yes, but it has occurred later (year? ....)
☐ Yes, in the past, but not now
☐ No, never
☐ Cannot be determined/Not applicable

Please provide examples:

a. If the description is true, did it occur suddenly, or gradually?
☐ Suddenly ☐ Gradually ☐ Don't know

b. How long have you exhibited this behavior?
☐ Only occasionally ☐ Less than 3 months
☐ 3 months–2 years ☐ Several years ☐ It varies

c. Is this behavior currently (the past 2 weeks) causing any problems?
☐ A lot ☐ Somewhat ☐ A little
☐ None at all ☐ Don't know

d. Has this behavior caused any problems in the past?
☐ A lot ☐ Somewhat ☐ A little
☐ None at all ☐ Don't know

---

30. Other symptoms can be listed below:

☐ Yes, always/as long as you can remember
☐ Yes, but it has occurred later (year? ....)
☐ Yes, in the past, but not now

Please provide examples:

a. If the description is true, did it occur suddenly, or gradually?
☐ Suddenly ☐ Gradually ☐ Don't know

b. How long have you exhibited this behavior?
☐ Only occasionally ☐ Less than 3 months
☐ 3 months–2 years ☐ Several years ☐ It varies

c. Is this behavior currently (the past 2 weeks) causing any problems?
☐ A lot ☐ Somewhat ☐ A little
☐ None at all ☐ Don't know

d. Has this behavior caused any problems in the past?
☐ A lot ☐ Somewhat ☐ A little
☐ None at all ☐ Don't know

---

31. What sort of problems due to health conditions have you experienced on average during the past 30 days? For each question, please circle one response.

	None	*Mild*	*Moderate*	*Severe*	*Extreme or cannot do*
1. Standing for long periods, such as 30 minutes?	☐	☐	☐	☐	☐
2. Taking care of your domestic responsibilities?	☐	☐	☐	☐	☐
3. Learning a new task, e.g., learning how to get to a new place?	☐	☐	☐	☐	☐
4. How much of a problem did you have joining community activities (e.g., festivities, religious or other activities) in the same way as anyone else can?	☐	☐	☐	☐	☐
5. How much have you been emotionally affected by your health problems?	☐	☐	☐	☐	☐
6. Concentrating on doing something for 10 minutes?	☐	☐	☐	☐	☐
7. Walking a long distance, such as a kilometer (or equivalent)?	☐	☐	☐	☐	☐
8. Washing your whole body?	☐	☐	☐	☐	☐
9. Getting dressed?	☐	☐	☐	☐	☐
10. Dealing with people you do not know?	☐	☐	☐	☐	☐
11. Keeping friends?	☐	☐	☐	☐	☐
12. Your day-to-day work?	☐	☐	☐	☐	☐

(Items from WHODAS 2.0.)

This questionnaire was filled out by:

☐ Patient ☐ Mother ☐ Father ☐ Sibling ☐ Spouse/partner ☐ Other: ....

Please provide name, address, E-mail, and phone number below for the person who responded to the questions:

Name ....

Address ....

Phone ....

E-mail..
