# Supplementary material for: Joint Hypermobility in Paediatric Acute-Onset Neuropsychiatric Syndrome—A Preliminary Case-Control Study
Source: Front Psychiatry. 2021 Dec 3;12:797165. doi: 10.3389/fpsyt.2021.797165 (PMC8678126; doi:10.3389/fpsyt.2021.797165)
Supplement: Data Sheet 2 — Clinician's interview. [file Data_Sheet_2.PDF]

Name: Date of birth: Date:

PNISSI

PsychoNeuroInflammatory related Signs and Symptoms Inventory

PNISSI is designed to facilitate a structured medical history for patients who have a presentation of atypical psychiatric symptoms and signs, which may indicate a neuroinflammatory process (such as might be the case with e.g. PANDAS and PANS; see summaries of these diagnostic criteria for these conditions at the end of this form). The symptoms included in PNISSI can thus occur in several neuroinflammatory conditions and likewise in "regular" psychiatric disorders.

PNISSI consists of five different sections: a self-report, a clinical interview, a motor skills and cognitive assessment (pages 13 and 15) and finally a clinical summary (page 19). Start with the self-report and end with the clinical summary. The order of the other sections is optional.

The clinical interview is based on the self-report, which the patient (or a related person) has filled out. The clinician is advised to read through their responses in advance.

The capital letter before each question is based on the corresponding question in the self-report. If you want to perform an abbreviated clinical interview, begin at item HH, page 5. If the questions are answered by a person other than the patient, then ”you” refers to the patient.

Gender: ☐ Female ☐ Male

Questions answered by:

☐ patient ☐ father ☐ mother ☐ other: ....

CLINICAL INTERVIEW

Questions A–F in the self-assessment, symptoms and course

1. At what age did your problems start? .... years
2. How did the symptoms appear at the beginning, and how have they developed since then?

....

....

....

....

....

....

....

....

....

....

....

....

3. At what age were the symptoms the worst, and what were they?

....

....

....

....

....

4. For how long did this period last, and when did you feel the worst?

....

5. Have you ever been free of symptoms? If yes, for how long did that period last?

....

....

....

6. Were there any events that may be related to the emergence of your problems e.g., infection (e.g. influenza/ dental infection), inflammation, travel, personal loss, strong frightening experience, vaccinations or other?

....

....

....

Symptom duration plot

The figures below depict possible courses of symptoms and/or progression of the problem. Show them to the person who you are interviewing and ask him/her to choose the most appropriate figure to describe the course. The horizontal axis depicts time, and the vertical axis depicts symptom severity. Please provide information on treatments and, if possible, the year and month of changes in symptoms and treatments. Different figures can be selected for different types of problems. You may use a separate sheet of paper and assist the person in charting the symptoms.

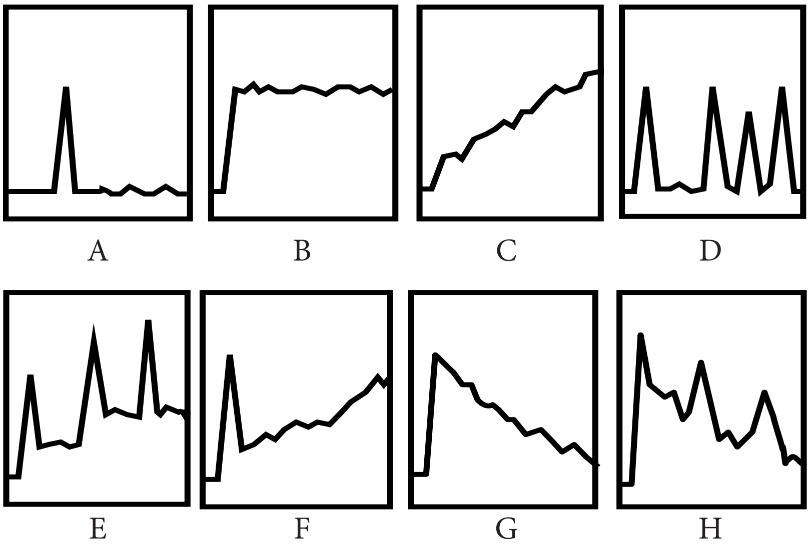

Flares are defined as new or worsening symptoms that develop over the course of hours or days and last at least 24 hours. Relapses usually last one to several weeks, and the symptoms remit fully or partly. **At least 30 days must pass between onset and relapse in order to distinguish them.**

7. If there is a relapsing course, how many relapses of OCD/hoarding/tics/anorexia/restricted eating/ other severe symptoms with a sudden onset (< 3 days) have you experienced? .... ☐ N/A
8. Are relapses related to infections or fever? (circle as applicable) ☐ Yes ☐ No ☐ Don't know
9. Have you noticed whether you improve or deteriorate when you have a fever? (circle as applicable) ☐ Yes ☐ No ☐ Don't know
10. Are relapses related to other factors? ☐ Yes ☐ No ☐ Don't know

    Comment:...
11. How old were you when you first sought help for your problems (e.g. for sleep disturbances, GLARE [difficult to comfort], eating problems, delayed psychomotor development, social problems, tics, or deviant language development)? .... years

    a. For which problems did you seek help? ....

    ....

    ....

    ....

    b. Do you still have these problems? ☐ Yes ☐ No ☐ Partly ☐ Don't know ☐ N/A

    ....

    ....
12. Are you worried about your physical health? ☐ Yes ☐ No ☐ Don't know

    a. If yes: What is your concern? ....

    ....

    ....

    b. When did you first become worried about your physical health?............ years of age (or what year?........)
13. Are you worried about your mental health? ☐ Yes ☐ No ☐ Don't know

    a. If yes: What is your concern? ....

    ....

    ....

    b. When did you first become worried about your mental health?............ years of age (or what year?........)

    c. When did you first become worried about the problems you present today? .... years of age.

    d. Did you receive a diagnosis at the time? ☐ Yes ☐ No ☐ Don't know

    e. If yes, which diagnosis/es? ....

    ....

    ....

Question G in the self-assessment

14. Do you have a physical illness or a physical diagnosis? ☐ Yes ☐ No ☐ Don't know

    a. If yes, which one(s), and at what age were you diagnosed?

    .... years

    .... years

15. Do you have a mental disorder or disability? ☐ Yes ☐ No ☐ Don't know

a. If yes, which one(s), and at what age were you diagnosed?

.... years

.... years

.... years

b. Do you have other problems that you presume should be diagnosed but where a formal diagnosis is lacking? ☐ Yes ☐ No ☐ Don't know

c. If yes, which, and at approximately what age did these symptoms/problems emerge?

.... years

.... years

.... years

Questions H–P in the self-assessment

16. If you have received treatment earlier, please specify the treatment (psychological, pharmaceutical, ECT, or other treatment) ☐ psychological ☐ pharmaceutical ☐ ECT ☐ other ☐ no treatment

....

....

....

a. If you had side-effects, what were they? ....

....

....

b. If medical treatment is ongoing, which medication(s) (regardless of which) and what dosage(s)?

....

....

....

c. If psychological or other treatment is presently ongoing, what kind of intervention is it?

....

....

d. How was the effect of the treatment(s) you received (pharmaceutical/psychological/other; what kind)?

The effect of treatment .... was ☐ obvious ☐ some ☐ no difference ☐ worse
☐ transient ☐ permanent ☐ not sure

The effect of treatment .... was ☐ obvious ☐ some ☐ no difference ☐ worse
☐ transient ☐ permanent ☐ not sure

The effect of treatment .... was ☐ obvious ☐ some ☐ no difference ☐ worse
☐ transient ☐ permanent ☐ not sure

The effect of treatment .... was ☐ obvious ☐ some ☐ no difference ☐ worse
☐ transient ☐ permanent ☐ not sure

Comment: ....

....

Question HH in the self-assessment

Go through the patient's/related person's responses in the self-report and specify endorsed symptoms – suggestions are shown below. Skip those questions that have been negated in the self-report (unless there is ambiguity in the patient's response or assuming the patient is dissimilating). Choose one response option per question. **An affirmative response corresponds to symptoms present during the past 2 weeks.** Circle specifications of present endorsed symptoms. To facilitate memorizing and illustrate connections, draw lines between specifications of endorsed symptoms and comments and underline relevant comments. The same comments could apply to various specifications.

Specification of endorsed symptoms	*Comments*

1. Obsessive/compulsive symptoms and hoarding

☐ Absolutely = 2 ☐ To some extent = 1 ☐ Previously = 0.5 ☐ No = 0 ☐ Don't know

a. excessive hand washing/fear of dirt/fear of contracting a disease
b. checking locks, checking doors, checking electrical appliances, checking that things have not been lost
c. requirements for a certain order or symmetry, ordering or repeating certain actions to reach "just the right feeling", or requiring others to repeat certain actions
d. hoarding
e. obsessions typical for OCD, such as fear of harming others

– has attracted attention from others
– more difficult to treat than expected
– not determined whether it is OCD related
– required psychiatric treatment
– relapse remitting course
– chronic course
– progressive course
– associated with general deterioration
– has ceased

2. Abnormal eating behavior

☐ Absolutely = 2 ☐ To some extent = 1 ☐ Previously = 0.5 ☐ No = 0 ☐ Don't know

a. loss of appetite
b. cannot force self to eat for unknown reasons
c. can only eat certain things (e.g. because of consistency, taste, smell)
d. sense of being overweight although being of normal weight
e. is related to obsessions with choking, throwing up, etc.
f. ideas that something inappropriate has been mixed in the food
g. increased appetite

– has required gavage/parenteral nutrition/inpatient care
– more difficult to treat than expected
– resulted in at least 10 % weight loss (.................. kg/lbs)
– resulted in weight gain (.................. kg/lbs)
– sudden weight gain for unknown reasons
– weight gain related to pharmaceutical treatment
– relapse remitting course
– chronic course
– progressive course
– associated with general deterioration
– has ceased

3. Separation anxiety

☐ Absolutely = 2 ☐ To some extent = 1 ☐ Previously = 0.5 ☐ No = 0 ☐ Don't know

a. cannot attend school (for periods longer than ..... weeks)
b. cannot go outside by him-/herself
c. can only be alone at home for short periods of time
d. cannot be alone at home
e. cannot be alone a room

– required psychiatric treatment
– more difficult to treat than expected
– specifically related to being bullied
– relapse remitting course
– chronic course
– progressive course
– associated with general deterioration
– has ceased

4. Depressed mood or significant mood swings

☐ Absolutely = 2 ☐ To some extent = 1 ☐ Previously = 0.5 ☐ No = 0 ☐ Don't know

a. sad for 2 weeks or more in a row
b. unexpected mood swings
c. depressive agitation
d. engaged in physical self-harm
e. expressed not wanting to live
f. attempted suicide

– fear that the person will hurt him-/herself
– relatives fear suicide
– more protracted than expected
– required psychiatric treatment
– required inpatient care
– relapse remitting course
– chronic course
– progressive course
– associated with general deterioration
– has ceased

5. Irritability

☐ Absolutely = 2 ☐ To some extent = 1 ☐ Previously = 0.5 ☐ No = 0 ☐ Don't know

a. sulks for prolonged periods of time about ”nothing”
b. has fits of rage
c. irritable most of the time
d. intimidating

– has attracted attention from others
– perceived as related to stress
– relapse remitting course
– chronic course
– progressive course
– associated with general deterioration
– has ceased

6. Oppositional defiant behavior

☐ Absolutely = 2 ☐ To some extent = 1 ☐ Previously = 0.5 ☐ No = 0 ☐ Don't know

a. often refuses to follow prompts
b. often irritates others purposely
c. often quick to fight back when somebody did something wrong
d. easily loses temper

– caused conflicts at home
– caused conflicts at school/work
– caused conflicts with friends/peers
– caused a virtually unbearable home situation
– relapse remitting course
– chronic course
– progressive course
– associated with general deterioration
– has ceased

7. Physically violent towards others

☐ Absolutely = 2 ☐ To some extent = 1 ☐ Previously = 0.5 ☐ No = 0 ☐ Don't know

a. has beaten others
b. has harmed others physically in other ways
c. has used weapons or equivalent

– family members or others have felt fear because of the outbreaks
– fear that the person will hurt someone
– not done with intent to harm but may result in harm
– outbreaks have caused serious conflicts
– has required police assistance or similar actions
– has been prosecuted/convicted/sentenced for violent behavior
– relapse remitting course
– chronic course
– progressive course
– associated with general deterioration
– has ceased

8. Physically violent towards oneself or objects

☐ Absolutely = 2 ☐ To some extent = 1 ☐ Previously = 0.5 ☐ No = 0 ☐ Don't know

a. has smashed or destroyed things on purpose
b. has hurt him-/herself

– family members or others have felt fear because of the outbreaks
– not done with intent to harm but may result in harm
– outbreaks have caused serious conflicts
– has required police assistance or similar actions
– has been prosecuted/convicted/sentenced for violent behavior
– relapse remitting course
– chronic course
– progressive course
– associated with general deterioration
– has ceased

---

9. Personality changes

☐ Absolutely = 2 ☐ To some extent = 1 ☐ Previously = 0.5 ☐ No = 0 ☐ Don't know

a. some changes compared with before onset
b. significantly altered (e.g., becomes intimidating)
c. appears as a stranger
d. behaves as though obsessed, feels like a horror movie

– has attracted attention from others
– scares family members
– occurs in bouts
– relapse remitting course
– chronic course
– progressive course
– associated with general deterioration
– has ceased

---

10. Abnormal psychomotor development (regression)

☐ Absolutely = 2 ☐ To some extent = 1 ☐ Previously = 0.5 ☐ No = 0 ☐ Don't know

a. loss of theoretical knowledge
b. loss of practical abilities
c. loss of language skills
d. behaves much younger than chronological age
e. baby-like behavior
f. marginal intellectual disability
g. intellectual disability

– probably an inborn abnormality
– probably an acquired abnormality
– relapse remitting course
– chronic course
– progressive course
– associated with general deterioration
– has ceased

---

11. Difficulties managing school or work

☐ Absolutely = 2 ☐ To some extent = 1 ☐ Previously = 0.5 ☐ No = 0 ☐ Don't know

a. impaired concentration
b. forgetful of what has been learned, impaired working memory
c. impared logical thinking

– has attracted attention from others
– cannot attend class anymore or unable to continue working
– attends special education
– relapse remitting course
– chronic course
– progressive course
– associated with general deterioration
– has ceased

12. Hypersensitivity to sensory input

☐ Absolutely = 2 ☐ To some extent = 1 ☐ Previously = 0.5 ☐ No = 0 ☐ Don't know

a. sensitive to sound
b. sensitive to light
c. sensitive to smells
d. sensitive to touch
e. sensitive to other sensory input:
....

– has attracted attention from others
– cannot attend class anymore because of hypersensitivity
– requires adjustments in everyday life
– relapse remitting course
– chronic course
– progressive course
– associated with general deterioration
– has ceased

13. Hallucinations

☐ Absolutely = 2 ☐ To some extent = 1 ☐ Previously = 0.5 ☐ No = 0 ☐ Don't know

a. visual hallucinations
b. hears mumbling or a thin voice
c. hears voices talking or commenting
d. senses taste/odors/smells
e. tactile hallucinations
f. senses others being in the room that cannot be seen

– has attracted attention from others
– is associated with anxiety
– requires adjustments in everyday life
– relapse remitting course
– chronic course
– progressive course
– associated with general deterioration
– has ceased

14. Perceptual abnormalities and misidentification

☐ Absolutely = 2 ☐ To some extent = 1 ☐ Previously = 0.5 ☐ No = 0 ☐ Don't know

a. feels that things are further away or closer than they really are
b. sense of having foreign body parts
c. senses bodyparts or items as enlarged or diminished
d. senses that things are floating around
e. sense of being dead (Cotard syndrome)
f. sense of other people having been replaced with impostors (Capgras syndrome)

– has attracted attention from others
– is associated with anxiety
– requires adjustments in everyday life
– relapse remitting course
– chronic course
– progressive course
– associated with general deterioration
– has ceased

15. Sense of being watched

☐ Absolutely = 2 ☐ To some extent = 1 ☐ Previously = 0.5 ☐ No = 0 ☐ Don't know

a. suspicious of others
b. suspicious of family members
c. feels persecuted
d. ideas of reference
e. paranoid delusions

– has attracted attention from others
– requires psychiatric care
– relapse remitting course
– chronic course
– progressive course
– associated with general deterioration
– has ceased

16. Confused behavior or incoherent speech

☐ Absolutely = 2 ☐ To some extent = 1 ☐ Previously = 0.5 ☐ No = 0 ☐ Don't know

a. cannot participate in a coherent conversation
b. incoherent speech
c. desorientated
d. mostly unresponsive
e. unresponsive

– has attracted attention from others
– requires psychiatric care
– most probably drug-induced
– relapse remitting course
– chronic course
– progressive course
– associated with general deterioration
– has ceased

17. Altered gaze

☐ Absolutely = 2 ☐ To some extent = 1 ☐ Previously = 0.5 ☐ No = 0 ☐ Don't know

a. looks terrified
b. looks violent
b. mydriasis (dilated pupils)

– has attracted attention from others
– relapse remitting course
– chronic course
– associated with general deterioration
– has ceased

18. Gross motor problems or muscle weakness

☐ Absolutely = 2 ☐ To some extent = 1 ☐ Previously = 0.5 ☐ No = 0 ☐ Don't know

a. clumsy movements
b. difficulties with coordination, apparent in e.g. ball games or dancing
c. aberrant gait
d. difficulty climbing stairs or walking on escalators
e. quadriceps and/or shoulder weakness when holding both arms straight out in front of the body for 60 seconds (noted in motor skills test)
f. paresis

– has attracted attention from others
– avoids participation in sports
– physically inactive
– percieved as a fixed idea
– relapse remitting course
– chronic course
– progressive course
– associated with general deterioration
– has ceased

19. Poor handwriting or difficulty sketching/drawing

☐ Absolutely = 2 ☐ To some extent = 1 ☐ Previously = 0.5 ☐ No = 0 ☐ Don't know

a. immature pen holding
b. deteriorated handwriting
c. deteriorated drawing/sketching
d. cannot write
e. cannot draw anything figuratively
f. better at drawing/sketching than writing

– has attracted attention from others
– refuses to draw/sketch
– illegible handwriting
– percieved as a fixed idea
– relapse remitting course
– progressive course
– chronic course
– associated with general deterioration
– has ceased

20. Involuntary movements or noises (tics)

☐ Absolutely = 2 ☐ To some extent = 1 ☐ Previously = 0.5 ☐ No = 0 ☐ Don't know

a. simple motor tics: rapid reflex-like intermittent movements
b. sequences of complex motor tics (may be difficult to separate from compulsions or stereotypies)
c. vocal tics
d. recurrent bouts of sound
e. other tics

– has attracted attention from others
– atypical age of onset of tics (e.g. post puberty)
– seems related to stress
– seems related to being relaxed
– lack of prodromal sensations
– most probably drug-induced
– relapse remitting course
– chronic course
– progressive course
– associated with general deterioration
– has ceased

21. Catatonia or mutism

☐ Absolutely = 2 ☐ To some extent = 1 ☐ Previously = 0.5 ☐ No = 0 ☐ Don't know

a. trapped in body movements (catalepsy, posturing)
b. speaks only with family members and not with others, i.e., selective mutism
c. speaks to nobody (but has speech), i.e., mutism
d. echolalia (echospeech)
e. echopraxia (imitation of movements)
f. automatism

– has attracted attention from others
– is associated with anxiety
– relapse remitting course
– chronic course
– progressive course
– associated with general deterioration
– has ceased

22. Involontary or unusual motor phenomenia

☐ Absolutely = 2 ☐ To some extent = 1 ☐ Previously = 0.5 ☐ No = 0 ☐ Don't know

a. complex movements ....
b. choreiform movements such as piano playing movement of fingers (noticeable in motor skill test)
c. choreoathetosis (i.e. involuntary movements in a combination of chorea (irregular migrating contractions) and athetosis (i.e. twisting and writhing)
d. dystonia (i.e. involuntary continual muscle contractions)
e. waxy flexibility (slight resistance to positioning by the examiner)
f. rigidity/parkinsonian symptoms
g. cogwheel rigidity
h. hitch in movement
i. stereotypies (mostly rhythmic e.g. head-banging, hand-flapping)
j. sudden jerks
k. ataxia
l. orofacial dyskinesia
m. seizures
n. oculogyric crisis
o. tremor ....

– has attracted attention from others
– seems related to stress
– seems related to being relaxed
– most probably drug-induced
– relapse remitting course
– chronic course
– progressive course
– associated with general deterioration
– has ceased

23. Urinary tract symptoms

☐ Absolutely = 2 ☐ To some extent = 1 ☐ Previously = 0.5 ☐ No = 0 ☐ Don't know

a. frequent urge to urinate
b. nervous of not making it to the bathroom when needed
c. enuresis in nighttime
d. enuresis in daytime

– has attracted attention from others
– assumed to be a fixed idea
– requires diapers
– relapse remitting course
– chronic course
– progressive course
– associated with general deterioration
– has ceased

24. Gut problems

☐ Absolutely = 2 ☐ To some extent = 1 ☐ Previously = 0.5 ☐ No = 0 ☐ Don't know

a. abdominal pain
b. diarrhea
c. constipation
d. both constipation and diarrhea
e. encopresis

– requires treatment
– more difficult to treat than expected
– requires daily adjustments
– requires inpatient care
– requires visit to emergency department
– most probably drug-induced
– relapse remitting course
– chronic course
– progressive course
– associated with general deterioration
– has ceased

25. Other pain-related symptoms

☐ Absolutely = 2 ☐ To some extent = 1 ☐ Previously = 0.5 ☐ No = 0 ☐ Don't know

a. muscle pain
b. joint pain
c. headache
d. migraine.
e. other pains ....

– requires recurrent treatment
– more difficult to treat than expected
– requires continuous treatment
– requires inpatient care
– relapse remitting course
– chronic course
– progressive course
– associated with general deterioration
– has ceased

26. Sleep disturbances

☐ Absolutely = 2 ☐ To some extent = 1 ☐ Previously = 0.5 ☐ No = 0 ☐ Don't know

a. insomnia
b. waking up during the night
c. restless sleep
d. night terrors
e. sleep paralysis
f. tired during the day
g. oversleeping
h. falling asleep unexpectedly during the day

– requires treatment
– more difficult to treat than expected
– has attracted attention from others
– most probably drug-induced
– relapse remitting course
– chronic course
– progressive course
– associated with general deterioration
– has ceased

27. Fatigue/exhaustion

☐ Absolutely = 2 ☐ To some extent = 1 ☐ Previously = 0.5 ☐ No = 0 ☐ Don't know

a. associated with stress
b. extreme exhaustion lasting more than 24 hours after physical or mental strain
c. extreme fatigue as in chronic fatigue syndrome

– swift recovery after rest
– requires daily adjustments
– relapse remitting course
– chronic course
– progressive course
– associated with general deterioration
– has ceased

28. Hyperactivity

☐ Absolutely = 2 ☐ To some extent = 1 ☐ Previously = 0.5 ☐ No = 0 ☐ Don't know

a. hyperactive signs, such as swinging the legs
b. moving all the time, constantly restless
c. speaks a lot, difficult to interrupt
d. runs back and forth
e. other...

– has attracted attention from others
– is associated with anxiety
– has required medication
– most probably drug-induced
– relapse remitting course
– chronic course
– progressive course
– associated with general deterioration
– has ceased

29. Abnormal vision

☐ Absolutely = 2 ☐ To some extent = 1 ☐ Previously = 0.5 ☐ No = 0 ☐ Don't know

a. vision loss
b. blurred vision
c. shadows in visual fields
d. dots in visual field

– has attracted attention from others
– associated with headache
– relapse remitting course
– chronic course
– progressive course
– associated with general deterioration
– has ceased

30. Other abnormalities

☐ Absolutely = 2 ☐ To some extent = 1 ☐ Previously = 0.5 ☐ No = 0 ☐ Don't know

Sum score *(range, 0–60)* ....

MOTOR SKILLS ASSESSMENT

Demonstrate and describe to the person how to perform each exercise.
If the patient is too young or refuses to perform the exercise, leave blank.

1. **TANDEM WALK**

Subject is asked to walk in a straight line for 12 feet (3 meters), heel to toe.

0 = no missteps after subject has completed first full step
1 = one or two missteps after completing first full step
2 = three or more missteps, grabbing to prevent falling

☐

2. **ROMBERG TEST**

Subject is asked to stand with his/her feet together, eyes closed, arms stretched out, parallel to the floor, with fingers spread apart. Maintain this position for 60 seconds.

0 = relatively stable, minimal swaying
1 = marked swaying
2 = subject steps to maintain balance and prevent falling

☐

3. **SHOULDER WEAKNESS**

Subject is asked to hold both arms straight out in front of the body, parallel to the floor (as in Romberg Test), and keep this position for 60 seconds.

0 = no problems
1 = tendency of arms dropping
2 = arms drop markedly

L ☐ R ☐

4. **ADVENTITIOUS OVERFLOW (PIANO FINGERS)**

Same as Romberg Test.

0 = no movement of fingers, hands, or arms
1 = irregular fluttering movements of fingers only
2 = irregular fluttering movements extended to hands and/or arms

L ☐ R ☐

5. **ALTERNATING SCISSOR JUMPS**

Subject is asked to assume a starting position with the right leg and left arm in front of the body and perform 15 rhythmic scissor jumps (i.e. alternating the opposite leg/arm in front of the body as if cross-country skiing).

0 = no difficulty and no hesitation
1 = some difficulty
2 = major difficulty or inability

☐

6. **RAPID ALTERNATING HAND MOVEMENTS**

Subject is asked to sit and place his/her hands palms down approximately 10 cm (4 in) proximally from the knees. The subject is asked to start with his/her dominant hand (usually the right hand) and slap his/her leg distinctly with the palm and back of his/her hand in an alternating motion. The subject is asked to perform the task rapidly 20 times, one hand at a time.

	L	R
0 = no major disruption of motion, no hesitation, no mistakes in hand placement<br>1 = one or two major disruptions of motion, hesitations, or mistakes in hand placement<br>2 = three or more major disruptions of motion, hesitations, or mistakes in hand placement	☐	☐

7. **MILKMAID'S GRIP**

The clinician faces the subject and holds the index and middle fingers of each hand pointing down like a cow's teats. The subject is asked to grab a "teat" with each of his/her hands and maintain a steady grip for 30 seconds.

0 = even grip
1 = slightly uneven grip
2 = a ”milking” motion of contraction and relaxation ☐

8. **DERMOGRAPHISM (SKIN WRITING)**

Subject is asked to remove shirt (or pull up sleeves and shirt). Lightly scratch the skin on the arms and torso with the back of a pen.

0 = no marks
1 = signs of marks
2 = scratches redden into a raised welt similar to hives ☐

9. **TONGUE PROTRUSION**

Subject is asked to stick out te tongue and keep it still for 30 seconds.

0 = no difficulty keeping it still
1 = slight tremor or minor movement
2 = frequent movements that seems to be involuntary ☐

Sum score *(range, 0–24* ....

---

10. **WEIGHT** _______

11. **HEIGHT** _______

12. **BLOOD PREASSURE** _______

13. **HEART RATE** _______

14. **TEMPERATURE** _______

COGNITIVE ASSESSMENT

Writing assignment

Copy the following sentences.

The dog is sitting in the court.

The cookies we received were very good.

Johnny thinks there are too many foxes in the woods around his house.

susanne.bejerot@oru.se

Drawing assignment 1

Copy the following shapes.

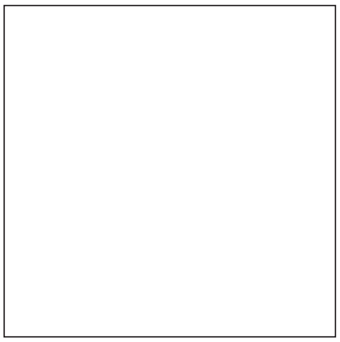

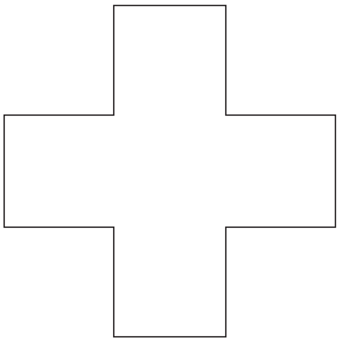

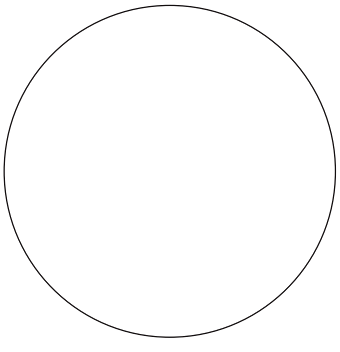

Drawing assignment 1

Copy the following shapes.

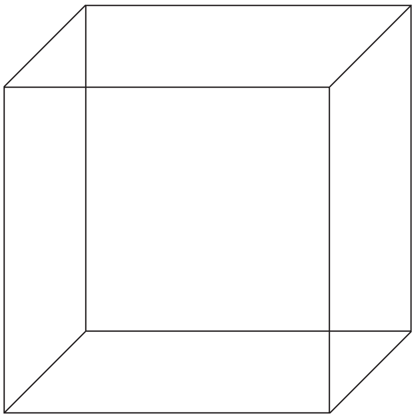

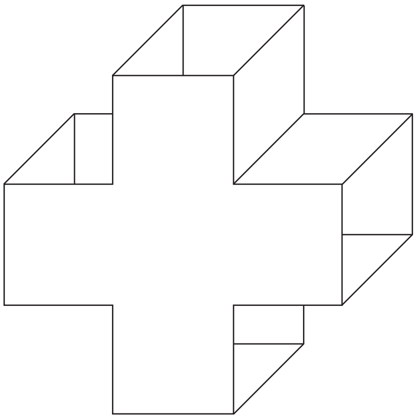

Drawing assignment 3

Fill in the numbers and hands to draw a clock showing ”a quarter to five”.

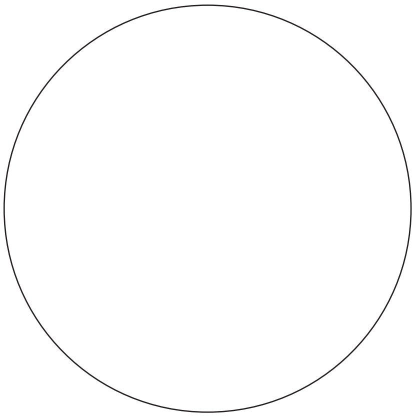

Drawing assignment 4

Draw a person (e.g., yourself). Use a separate sheet of paper.

GLOBAL SEVERITY – MENTAL SYMPTOMS

(include cognitive symptoms) Current

a. Severity of current psychiatric symptoms (exclude any preexcisting intellectual disability if present) ....

b. Severity of regression/lost abilities ....

c. Severity of personality changes ....

d. Severity of intellectual disability ....

- Not assessed	*4 Moderate*
1 None	*5 Marked*
2 Borderline	*6 Severe*
3 Mild	*7 Extremely severe*

Severity of current *psychiatric symptoms,* **sum score** *(range 4–28)*

GLOBAL SEVERITY – PHYSICAL SYMPTOMS

(include disordered sleep, appetite, pain, motor skills and fatigue) Current

a. Severity of present index symptoms (exclude physical symptoms that were present already before onset) ....

b. Severity of acquired motor skills problems ....

c. Severity of sleep disturbances ....

d. Severity of fatigue ....

- Not assessed	*4 Moderate*
1 None	*5 Marked*
2 Borderline	*6 Severe*
3 Mild	*7 Extremely severe*

Severity of current *physical symptoms,* **sum score** *(range 4-28)* ....

Overall Clinical Global Impression -Severity, CGI-S (1-7), current ....

Overall Clinical Global Impression -Severity, CGI-S (1-7), ever ....

Overall Clinical Global Impression - Improvement, CGI-I (1-7)

A. ☐ since worst symptoms (mm/yy)......./.......

B. ☐ since last visit (mm/yy)......../.......

- Not applicable/unknown	*4. No change*
1. Very much improved	*5. Minimally worse*
2. Much improved	*6. Much worse*
3. Minimally improved	*7. Very much worse*

CLINICAL SUMMARY

SUM SCORES (SCORES ADDED FROM PAGES 12, 14 OCH 18, RANGE 8-140) ☐

Acute or sub acute onset

☐ < 24 hrs ☐ 1–3 days ☐ 4–30 days ☐ 1–3 months ☐ > 3 months–12 months ☐ > 1 year

Focal neurological symptoms: paresis, ataxia, dystonia, chorea / athetosis, myoclonus, sensory loss, paresthesias, cranial nerve affection, sphincter dysfunction; also subtle motor symptoms that are new, such as impaired ability to write ☐ Yes ☐ No ☐ Unknown

Psychiatric symptoms: OCD or anorexia with unusually abrupt onset or psychosis with atypical features ☐ Yes ☐ No ☐ Unknown

Seizures ☐ Yes ☐ No ☐ Unknown

Dysautonomia: hyper/hypotension, tachycardia/bradycardia, hypoventilation, improper temperature control ☐ Yes ☐ No ☐ Unknown

Course

- ☐ Flares with an initial return to normal initially but thereafter gradual deterioration with each flare
- ☐ Progressive course with gradual deterioration without obvious flares
- ☐ Flares with recovery to normal
- ☐ Chronic/stable
- ☐ Chronic progressive with flares
- ☐ Gradual improvement
- ☐ Stable recovery back to normal (since mm/yy ....)

Related to infection or fever (parainfectious or postinfectious)

Onset related to strep infection?	☐ Confirmed	☐ Likely	☐ No	☐ Unknown
Onset related to throat problems?	☐ Confirmed	☐ Likely	☐ No	☐ Unknown
Onset related to other infection?	☐ Confirmed	☐ Likely	☐ No	☐ Unknown
....	☐ Confirmed	☐ Likely	☐ No	☐ Unknown
**Headache, vomiting, stiff neck, impaired general condition**	☐ Confirmed	☐ Likely	☐ No	☐ Unknown

Related to inflammation

Has a defined inflammatory disease?	☐ Confirmed	☐ Likely	☐ No	☐ Unknown
Improvement when having a fever?	☐ Confirmed	☐ Likely	☐ No	☐ Unknown
Improvement from cortisone?	☐ Confirmed	☐ Likely	☐ No	☐ Unknown
Improvement from NSAIDs?	☐ Confirmed	☐ Likely	☐ No	☐ Unknown
Improvement from antibiotics?	☐ Confirmed	☐ Likely	☐ No	☐ Unknown
Improvement from other treatment?	☐ Confirmed	☐ Likely	☐ No	☐ Unknown

Reliability of the assessment as a whole

0. Excellent, no reason to suspect data is unreliable
1. Good, factor(s) present that may adversely affect reliability
2. Fair, factor(s) present that definitely reduce reliability
3. Poor, very low reliability

Criteria for some relevant diagnoses

PANDAS<br>Pediatric autoimmune neuropsychiatric disorders associated with streptococcal infections (Swedo et al, Am J Psychiatry 1998, 155(2): 264–271)	PANS<br>Pediatric acute-onset neuropsychiatric syndrome (Swedo et al, Pediatrics & Therapeutics 2012, 2(2))	Idiopathic CANS<br>Childhood acute neuropsychiatric symptoms (Singer et al, J Pediatr 2012, 160(5): 725–731)
All five diagnostic criteria must be met:<br>I. Presence of obsessive-compulsive disorder (OCD) or a tic disorder.<br>II. Prepubertal symptom onset and after age of 3 years.<br>III. Acute symptom onset and episodic (relapsing-remitting) course.<br>IV. Temporal association between Group A streptococcal infection and symptom onset/exacerbations.<br>V. Associated with neurological abnormalities (particularly motoric hyperactivity and choreiform movements).	I. Abrupt, dramatic onset of obsessive-compulsive disorder or severely restricted food intake.<br>II. Concurrent presence of additional neuropsychiatric symptoms, with similarly severe and acute onset, from at least two of the following seven categories (see text for full description):<br>1. Anxiety.<br>2. Emotional lability and/or depression.<br>3. Irritability, aggression, and/or severely oppositional behaviors.<br>4. Behavioral (developmental) regression.<br>5. Deterioration in school performance.<br>6. Sensory or motor abnormalities.<br>7. Somatic signs and symptoms, including sleep disturbances, enuresis or urinary frequency.<br>III. Symptoms are not better explained by a known neurologic or medical disorder, such as Sydenham chorea, systemic lupus erythematosus, Tourette disorder, or others.	Acute dramatic onset of neuropsychiatric symptoms before 18 years of age.<br><br>I. Primary criterion: OCD.<br>II. Secondary criteria: Tics, dysgraphia, clumsiness, hyperactivity, anxiety, psychosis, developmental regression, sensitivity to sensory stimuli, emotional lability.<br>III. Either mono- or polyphasic.<br><br>The proposed CANS classification does not require association with a specific organism, limitation of symptoms to tics or OCD, a specific age range, or recurrence of symptoms. It is unknown at this time whether this idiopathic category will have definable clinical features or characteristic laboratory findings.

Current diagnoses: Rank the present diagnoses: primary (1), secondary (2), tertiary (3) etc

☐ ADHD	☐ DCD (Clumsiness)	☐ **IBD Inflammatory Bowel Disease**	☐ PANS/CANS
☐ Anorexia	☐ Depression	☐ **IBS Inflammatory Bowel Syndrome**	☐ **POTS**
☐ ARFID, Avoidant restricted food intace	☐ Dissociative disorder	☐ **Narcolepsy**	☐ Psychosis NOS
☐ ASD, Autism spectrum disorder	☐ Emotionally unstable personality	☐ **Obesity**	☐ **Rheumatic disease**
☐ Anxiety NOS	☐ **Encephalitis**	☐ OCD	☐ Schizophrenia
☐ Bipolar	☐ **Epilepsy**	☐ Oppositional defiant	☐ Separation anxiety
☐ Catatonia/Mutism	☐ Hoarding	☐ **Pain disorder**	☐ Self-harm (NSSID)
☐ **Chronic fatigue syndrome**	☐ ID, Intellectual disability	☐ PANDAS	☐ Tics

Comments:

....

....

....

....
